# Evolutionary analysis of the female-specific avian W chromosome

**DOI:** 10.1038/ncomms8330

**Published:** 2015-06-04

**Authors:** Linnéa Smeds, Vera Warmuth, Paulina Bolivar, Severin Uebbing, Reto Burri, Alexander Suh, Alexander Nater, Stanislav Bureš, Laszlo Z. Garamszegi, Silje Hogner, Juan Moreno, Anna Qvarnström, Milan Ružić, Stein-Are Sæther, Glenn-Peter Sætre, Janos Török, Hans Ellegren

**Affiliations:** 1 Department of Evolutionary Biology, Evolutionary Biology Centre, Uppsala University, Norbyvägen 18D, SE-752 36 Uppsala, Sweden; 2 Laboratory of Ornithology, Department of Zoology, Palacky University, 77146 Olomouc, Czech Republic; 3 Department of Evolutionary Ecology, Estación Biológica de Doñana-CSIC, 41092 Seville, Spain; 4 Department of Biosciences, Centre for Ecological and Evolutionary Synthesis, University of Oslo, 0316 Oslo, Norway; 5 Natural History Museum, University of Oslo, 0318 Oslo, Norway; 6 Museo Nacional de Ciencias Naturales-CSIC, 28006 Madrid, Spain; 7 Department of Animal Ecology, Evolutionary Biology Centre, Uppsala University, 75236 Uppsala, Sweden; 8 Bird Protection and Study Society of Serbia, Radnička 20a, 21000 Novi Sad, Serbia; 9 Norwegian Institute for Nature Research (NINA), 7034 Trondheim, Norway; 10 Behavioural Ecology Group, Department of Systematic Zoology and Ecology, Eötvös Loránd University, 1117 Budapest, Hungary

## Abstract

The typically repetitive nature of the sex-limited chromosome means that it is often excluded from or poorly covered in genome assemblies, hindering studies of evolutionary and population genomic processes in non-recombining chromosomes. Here, we present a draft assembly of the non-recombining region of the collared flycatcher W chromosome, containing 46 genes without evidence of female-specific functional differentiation. Survival of genes during W chromosome degeneration has been highly non-random and expression data suggest that this can be attributed to selection for maintaining gene dose and ancestral expression levels of essential genes. Re-sequencing of large population samples revealed dramatically reduced levels of within-species diversity and elevated rates of between-species differentiation (lineage sorting), consistent with low effective population size. Concordance between W chromosome and mitochondrial DNA phylogenetic trees demonstrates evolutionary stable matrilineal inheritance of this nuclear–cytonuclear pair of chromosomes. Our results show both commonalities and differences between W chromosome and Y chromosome evolution.

## Abstract (web summary)

The evolution of non-recombining chromosomes is poorly understood. Here, the authors sequence the collared flycatcher female-specific W chromosome and show nonrandom survival of genes during W chromosome degeneration which is due to selection for maintaining gene dose and expression levels of essential genes.

## Graphical abstract

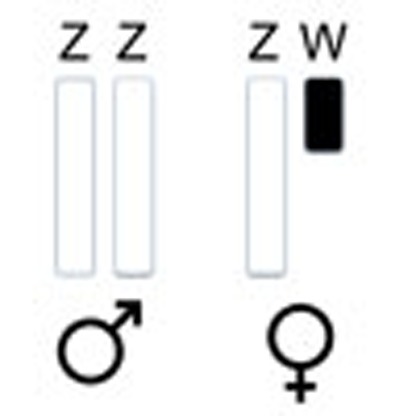

The nuclear genome of sexually reproducing animals is shared between males and females and is thus affected by evolutionary processes pertinent to both sexes. This comes with the exception of the non-recombining part of the sex-limited chromosome, that is, the Y chromosome in male heterogametic organisms (females XX, males XY) and the W chromosome in female heterogametic organisms (females ZW, males ZZ), where sequence evolution reflects processes specific to one sex. This has been utilized in Y chromosome-based molecular evolutionary studies in *Drosophila*^1^ and humans^2^, and has provided a paternal view of demography and migration in human populations^3^. Like mammalian and other Y chromosomes^4^,^5^, avian W chromosomes are in most cases highly heterochromatic and degenerated variants of once recombining proto-sex chromosomes^6^,^7^, aggravating sequence assembly and downstream analyses. In the chicken two satellite DNA repeat families alone are estimated to correspond to ≈75% of the W chromosome, with other amplified repeat families contributing to the remaining sequence^8^.

A small effective population size (*N*_e_) and the sensitivity to selection that follows from absence of recombination and exposure of recessive mutations should be common features of both Y and W chromosomes. However, there might also be differences between the two types of sex-limited chromosomes^9^. For example, sexual selection, acting as a potent force on the evolution of male-specific, Y-linked genes^10^,^11^, should have a negligible effect on W chromosome evolution. Moreover, transmission through oogenesis rather than spermatogenesis implies that W chromosomes are exposed to a different mutational and epigenetic germ line environment than Y chromosomes. How these and other factors affect W chromosome evolution are largely unknown and the lack of large-scale polymorphism data has hindered population genomic analyses of W-linked sequences. Here, we study W chromosome evolution in four black-and-white flycatchers of the genus *Ficedula*, which are ecological model species for studies of life history evolution, speciation and mating systems^12^. We find no evidence for functional differentiation of the 46 genes identified on the W chromosome, all of which have a gametologous copy on the Z chromosome. Rather than representing a random set of genes surviving on the degenerating W chromosome, selection has independently preserved the gene content of the W chromosome in different avian lineages, potentially driven by dosage sensitivity. We find that neutral W-linked sequences evolve slowly because of male-biased mutation but that slightly deleterious mutations accumulate at a high rate due to a low effective population size. Because of the latter, nucleotide diversity is low and the rate of lineage sorting high on the W chromosome. Finally, we demonstrate complete matrilineal co-inheritance of the W chromosome and mitochondrial DNA.

## Results

### Gene content of the W chromosome

By making a *de novo* genome assembly from female DNA and subsequently mapping male and female re-sequencing reads to identify W-specific contigs with very stringent criteria (see Methods), we assembled 6.9 Mb of sequence from the non-recombining region of the W chromosome (NRW) of the collared flycatcher, *Ficedula albicollis* (Supplementary Table 1, Supplementary Fig. 1). The assembly likely represents a large part of the euchromatic region of the W chromosome. Transposable elements (TE) were embedded within NRW sequence to a much larger extent than elsewhere in the flycatcher genome (TE density on NRW=48,5%, Z chromosome=8.8% and autosomes=5.9%; Supplementary Table 2). The repertoire of repeat categories differed significantly between the NRW and the rest of the genome, with an overrepresentation of long copies of chicken repeat 1 (CR1) elements and potential full-length retroviral elements (Supplementary Fig. 2).

The NRW assembly contained 46 protein-coding genes (Table 1) and five pseudogenes. A comparison with the gene content of the flycatcher Z chromosome revealed that all NRW genes have a gametologous (that is, a non-recombining paralogue) copy on the Z chromosome. This demonstrates the common origin of the Z and W chromosomes from an ancestral pair of autosomes, before sex chromosome divergence, and provides an independent validation of that the identified W-linked genes are indeed located on the W chromosome. There was thus no evidence for transposition of genes to the W chromosome from autosomes. This is in contrast to the situation for mammalian and *Drosophila* Y chromosomes where the acquisition of genes involved in male reproduction is suggested to be driven at least in part by sexual selection^13^,^14^,^15^. Mechanistically, the absence of LINE-1 retrotransposons recognizing poly-A tails of mRNAs^6^ in avian genomes should render gene transpositions rare in birds.

Only one out of the 46 NRW genes was multi-copy, *HINTW*, as evidenced by unusually high sequence coverage resulting from collapsed mapping of slightly divergent copies. *HINTW* is the only ampliconic gene found on avian W chromosomes, with up to 40 copies observed in chicken^16^ and with evidence for gene conversion (intrachromosomal recombination) leading to concerted evolution among gene copies within species^17^. Ampliconic genes are more common on mammalian Y chromosomes but are mainly restricted to recently amplified gene families in regions acquired to the Y chromosome subsequent to cessation of recombination between sex chromosomes^18^. In ancestral parts of Y chromosomes, ampliconic genes may be as rare as they apparently are in avian W chromosomes^14^.

There was a distinctly lower GC content of NRW genes (mean GC3=38.14%±1.44 s.e.m.) than of Z-linked gametologs (46.17±2.25%; *P*<0.001, Wilcoxon signed-rank test), which is in line with predictions from a GC-biased gene conversion model where GC content should be higher for a recombining than for a non-recombining sequence.

We found no evidence for an overrepresentation of annotated functions or processes related to female reproduction among NRW genes (Supplementary Table 3). In contrast, an enrichment of testis-specific genes is observed on the Y chromosome in male heterogametic organisms^13^. Moreover, the expression of NRW genes was less pronounced in ovaries (on average, 10.2% of total expression across eight tissues was detected in ovaries) than it was for their Z-linked gametologs (13.3%, *P*=0.004) and other Z-linked genes (17.4%; *P*=0.02, Mann–Whitney *U*-test). Moreover, ovary expression levels were significantly lower for NRW genes (median=0.405 zFPKM) than for their Z-linked gametologs (0.712, *P*=0.00003, Wilcoxon signed-rank test) and similar to that of other Z-linked genes (0.443, *P*=0.49, Mann–Whitney *U*-test). Female expression levels of W-linked and Z-linked gametologs were highly correlated in all examined tissues (Supplementary Fig. 3). Furthermore, the expression profiles across tissues were highly similar between gametologs (Supplementary Fig. 4). Overall, this indicates the absence of functional differentiation of NRW genes subsequent to recombination restriction and does not support a role of the W chromosome in female fertility.

We found that the character of expression of Z-linked genes with a gametologous copy on the W chromosome differed from that of Z-linked genes without a gametologous copy in two respects. First, genes with a W-linked gametolog were more broadly expressed (mean *τ* measured in males=0.45±0.18; a *τ* of 1 means tissue-specific expression) than genes without a W-linked gametolog (mean *τ*=0.66±0.20; *P*<10^−9^). Second, the inter-individual variance was generally smaller, that is, more tightly regulated expression level, of Z-linked genes with a retained W-copy than of Z-linked genes without a retained W-copy (*P*<0.0001 in skin and kidney, *P*<0.05 in brain, liver and muscle, *P*>0.05 in lung testis and embryo; Supplementary Table 4).

### Gene expression in relation to gene dosage

**Figure 1:**
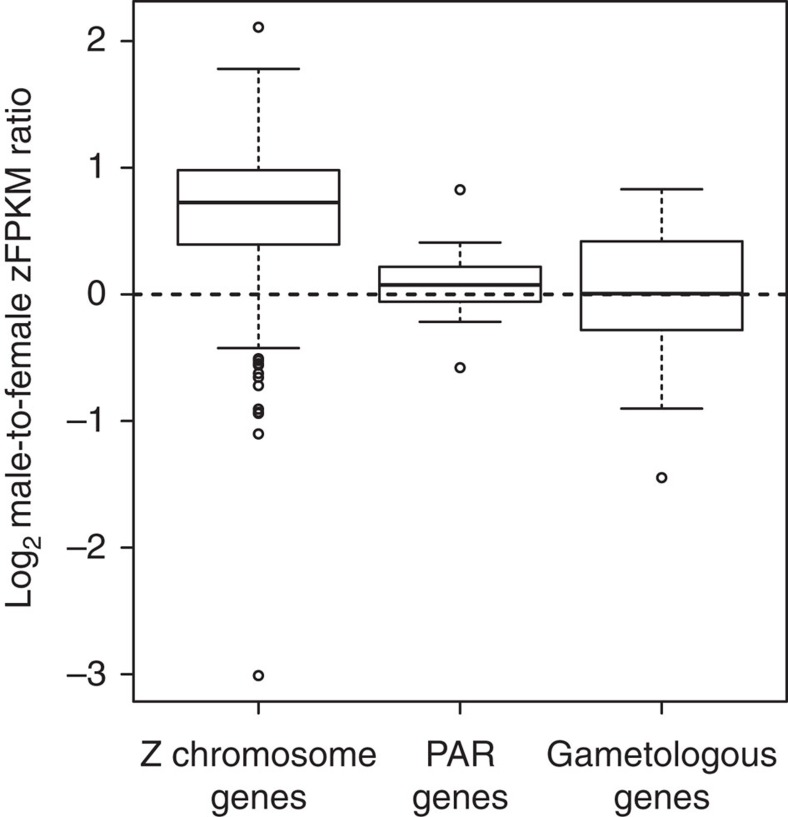
Male-to-female expression ratios (log_2_) for genes on the collared flycatcher sex chromosomes. Box plots of mean values over seven different tissues are shown. Left, genes from the Z chromosome without a W-linked copy (*n*=600; ref. 22); middle, genes from the small (630 kb) pseudoautosomal region (PAR; *n*=20; ref. 21); right, gametologous gene pairs (*n*=44). Boxes show distribution quartiles with the median in bold. Whiskers show minimum and maximum of the distribution unless this is more than 1.5 times the interquartile distance. Outliers exceed this limit.

Avian dosage compensation of sex-linked genes is incomplete, with male expression of Z-linked genes without a W-linked gametolog (that is, the vast majority of genes on the avian Z chromosome) being on average ≈1.5 times higher than female expression^19^,^20^ and with some genes showing equal expression in the two sexes. If W-linked gametologs are not generally functionally differentiated, their expression could serve as a means for females to maintain ancestral expression levels of critical genes after cessation of recombination between the Z and W chromosome. We compared male and female expression levels of gametologous gene pairs and could confirm this hypothesis: male Z+Z and female Z+NRW expression levels were almost equal for most gametologous genes (Fig. 1, Supplementary Fig. 5), similar to the situation for genes in the minute pseudoautosomal region (PAR) of flycatcher sex chromosomes^21^. Interestingly, the expression level of the single Z chromosome in females (mean across tissues=2.84 zFPKM±0.19) was generally higher, and that of the W chromosome (1.14±0.044) generally lower, than the per-Z chromosome expression of males (total male Z+Z expression: 4.02±0.12). In a scenario of selection for maintaining ancestral levels in females, this could be because the halved Z-linked gene dose in females does not translate into halved expression level and expression of NRW genes is adjusted (downwards) accordingly. Alternatively, Z-linked expression in females may be adjusted upwards (as is done for most Z chromosome genes without a W gametolog^22^; Fig. 1) to compensate for reduced W-linked gene expression resulting from NRW degeneration. Higher expression of the Z-linked than of the W-linked gametolog has been observed in some other bird species^7^. In ostrich, a species in which dosage compensation seems essentially absent, the combined expression of Z-linked and W-linked gametologs in females also result in equal male Z+Z and female Z+NRW expression levels for some but not all sex-linked genes^23^.

### Non-random decay of genes from the W chromosome

**Figure 2:**
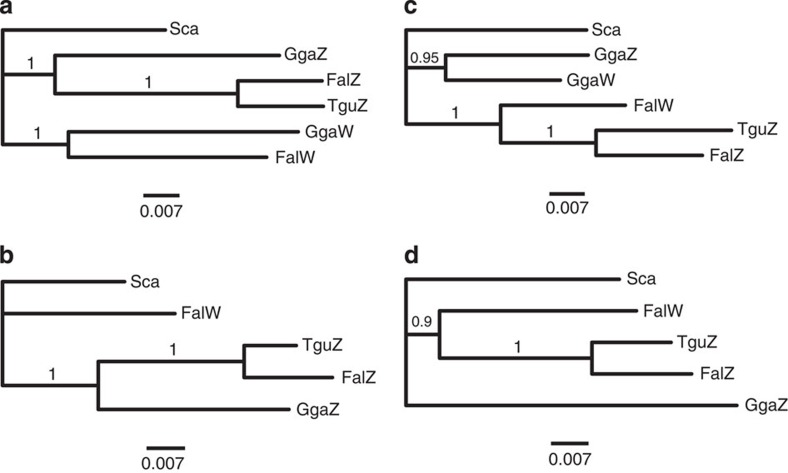
Examples of phylogenetic trees of gametologous gene pairs demonstrating the presence of at least two evolutionary strata on the flycatcher Z chromosome. (**a**) *CHD1*, (**b**) *GNAQ*, (c) *VCP* and (d) *ZNF131*. **a** and **b** are genes where Z-W recombination ceased prior to the split of flycatcher and chicken lineages, meaning that genes cluster by gametologs. (**c**) and (**d**) are genes where Z–W recombination ceased subsequent to the split of flycatcher and chicken lineages, meaning that genes cluster by species. **a** and **d** are examples of genes where chickens lacks a known W-linked gametolog. Species codes: Sca, *Struthio camelus* (ostrich); Gga, *Gallus gallus* (chicken); Tgu *Taeniopygia guttata* (zebra finch); Fal, *Ficedula albicollis* (collared flycatcher).

All gametologous flycatcher genes are located on the Z chromosome in chicken, reflecting the high degree of synteny conservation in birds^24^. Some of these genes also have a gametologous W-linked copy in chicken identified by microarray analysis or RNA-seq in the absence of a comprehensive chicken NRW assembly^25^,^26^,^27^. Analysis of the phylogenetic relationships among homologous genes revealed one group that clustered by species, that is, [flycatcher Z, flycatcher W][chicken Z, chicken W (if present)], and one group that clustered by chromosome [flycatcher Z, chicken Z][flycatcher W, chicken W (if present)] (Fig. 2, Supplementary Table 5). These categories correspond to at least two different evolutionary strata, similar to what has been seen in previous avian work^7^,^25^,^28^,^29^,^30^, with the latter representing genes from the avian proto-sex chromosomes that ceased to recombine before the split of the lineages leading to chicken (order Galliformes within the clade Galloanserae, one of the two major lineages of Neognath birds) and flycatcher (order Passeriformes within Neoaves, which is the other major Neognath lineage) 90 million years ago (myr ago), and the former genes where recombination arrest was initiated independently in the two lineages after their split. As expected, mean sequence divergence between gametologs was higher for genes in the ‘old’ stratum (0.330±0.019) than in the ‘young’ stratum (synonymous substitution rate, *d*_S_, 0.262±0.024), although we notice that there was considerable overlap in *d*_S_ estimates at the level of individual genes between the two categories (Supplementary Table 5). Because of the latter, we suggest that it may be more difficult than previously acknowledged to assign individual genes to particular evolutionary strata, or define the precise borders between strata, based on divergence data alone. Zhou *et al*.^7^ have recently provided a detailed portray over the emergence of evolutionary strata across divergent bird lineage. Their study did not contain a representative of the order Passeriformes (which split from other Neoavian lineages 55 myr ago, in connection with an extremely rapid adaptive radiation of Neoavian lineages^31^), so we cannot use the phylogenetic approach to test whether the ‘young’ flycatcher stratum is composed of two or more distinct strata.

For the purpose of this study, definition of evolutionary strata are relevant in the context of studying the survival/loss of genes that independently have ceased to recombine in flycatcher and chicken lineages. Genes belonging to the young stratum are spread from position 1.4 to 27.1 Mb on the flycatcher Z chromosome (≈45% of the chromosome). This chromosome segment contains a total of 277 genes shared between flycatcher and chicken (the two species’ Z chromosomes are co-linear in this region^32^), with 19 of these surviving on the flycatcher NRW and 17 on the chicken NRW. If survival by resistance to degeneration on the avian NRW were random processes in the two independent lineages, we would have expected one (1.17) gene to be common to the two species’ list of surviving genes. However, a vast excess of 12 such genes was observed (*P*<10^−8^), suggesting a highly non-random process of gene survival on the avian W chromosome after the arrest of recombination. The test is conservative since additional genes might be found to be common when the chicken NRW sequence gets assembled. Together with the observations of similar expression profiles of gametologous gene pairs, similar Z+Z and Z+NRW expression levels and absence of functional differentiation of W-linked gametologs, we suggest that selection has favoured the retention of tightly regulated, dosage-sensitive genes on degenerating avian W chromosomes. This mirrors the situation for a conserved group of regulatory genes on mammalian Y chromosomes^27^,^33^.

### Mutation and selection on the W chromosome

Gametologous gene pairs provide a natural experiment for molecular evolutionary analyses of gene sequences in relation to sex and recombination environment. The neutral rate of sequence evolution of W-linked genes specifically reflects the female mutation rate and the extent to which protein evolution is affected by selection reflects selection operating on females only in a non-recombining chromosome. Lineage-specific *d*_*S*_ since cessation of recombination was on average 1.57 (±0.13; median 1.62) times higher for the Z-linked than for the W-linked copy of gametologous pairs, corresponding to a male-to-female mutation rate ratio of 1.93 (Supplementary Table 5). This is similar to point estimates obtained from analyses of individual genes in different bird species^34^ and demonstrates that the avian W chromosome has a uniquely low rate of germ line mutation. Substitution rate estimates corroborated the expectation of reduced efficacy of purifying selection in non-recombining chromosomes^5^, manifested in significantly higher ratios of the rates of non-synonymous to synonymous substitution (*d*_N_/*d*_S_) for W-linked genes (mean=0.192±0.040) than for their Z-linked gametologs (mean=0.062±0.018; *P*=0.00018, Wilcoxon signed-rank test; Supplementary Table 5), similar to what has been seen in other bird species^7^,^35^.

### Population genomics of the W chromosome

**Figure 3:**
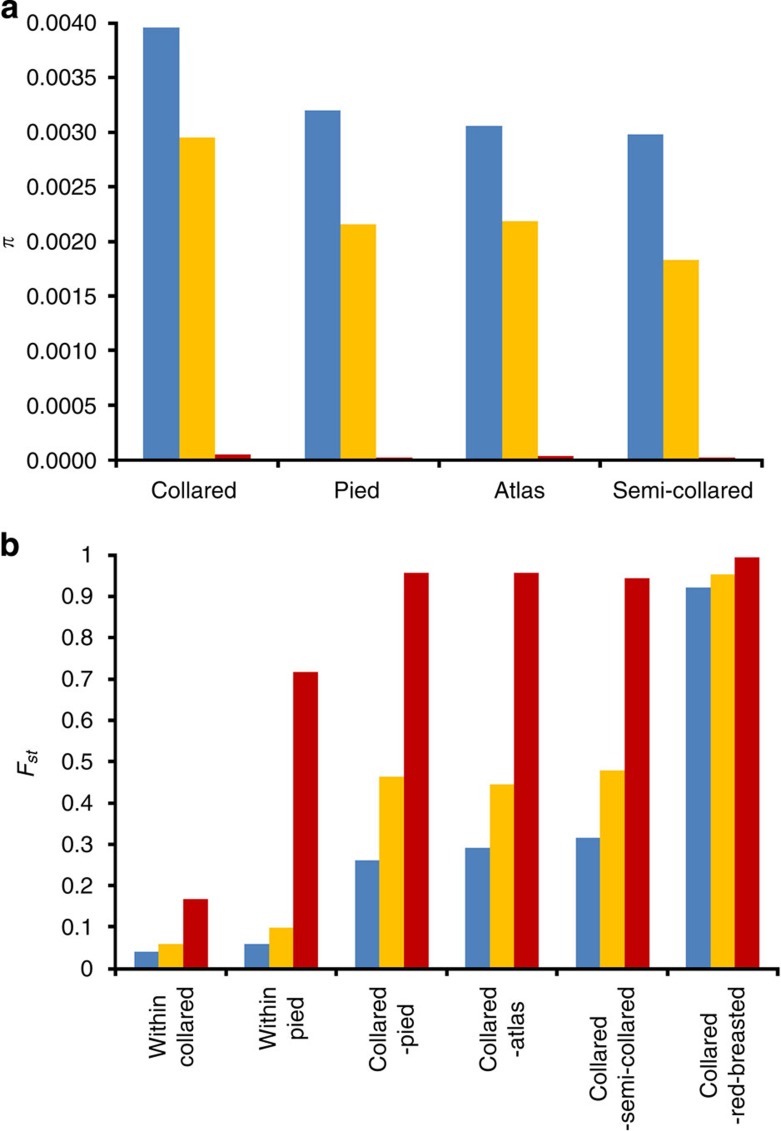
Population genomics of NRW sequences. (**a**) Pairwise nucleotide diversity (*π*) for different *Ficedula* species, showing drastically reduced levels of diversity on the NRW. (**b**) Degree of genetic differentiation (*F*_*st*_) between different *Ficedula* populations (within collared flycatcher and pied flycatcher, respectively) and species (collared flycatcher versus each of the three other black-and-white flycatcher species and the outgroup species red-breasted flycatcher). Colour codes: red, NRW; yellow, Z chromosome; blue, autosomes.

Population genomic analyses of sex-limited chromosomes are rare and have, to our knowledge, not been reported for female heterogametic organisms. We re-sequenced the genomes of 96 females from multiple populations of four closely related flycatcher species (besides collared flycatcher also pied flycatcher *F. hypoleuca*, semi-collared flycatcher *F. semitorquata* and Atlas flycatcher *F. speculigera*) and an outgroup (red-breasted flycatcher *F. parva*), mapped reads to the collared flycatcher reference genome^32^,^36^ as well as the new NRW assembly, and called variant sites. Nucleotide diversity on the NRW was drastically lower than in the rest of the genome (Fig. 3a, Supplementary Table 6), with a mean number of pairwise differences within species of 3.8−6.9 × 10^−5^ per bp (autosomes: 2.97−3.97 × 10^−3^). Taking the lower mutation rate and the equilibrium neutral expectation of one-quarter the autosomal level of diversity into account, genetic diversity of the W chromosome was 8–13 times lower than that of autosomes (Supplementary Table 6). This reduction of diversity is at least as pronounced as that observed for the human Y chromosome, which has 5–10 lower diversity than autosomes^37^. Although sexual selection might shape diversity levels of Y chromosomes via selective sweeps in testis-specific genes^38^, our data demonstrate significant loss of diversity in non-recombining sex chromosomes even in the likely absence of sexual selection. Nevertheless, selection is the most viable explanation to reduced NRW diversity given by the observation of a shift towards rare alleles in the unfolded site frequency spectrum of NRW sequences compared with autosomal sequences (Supplementary Fig. 6).

**Figure 4:**
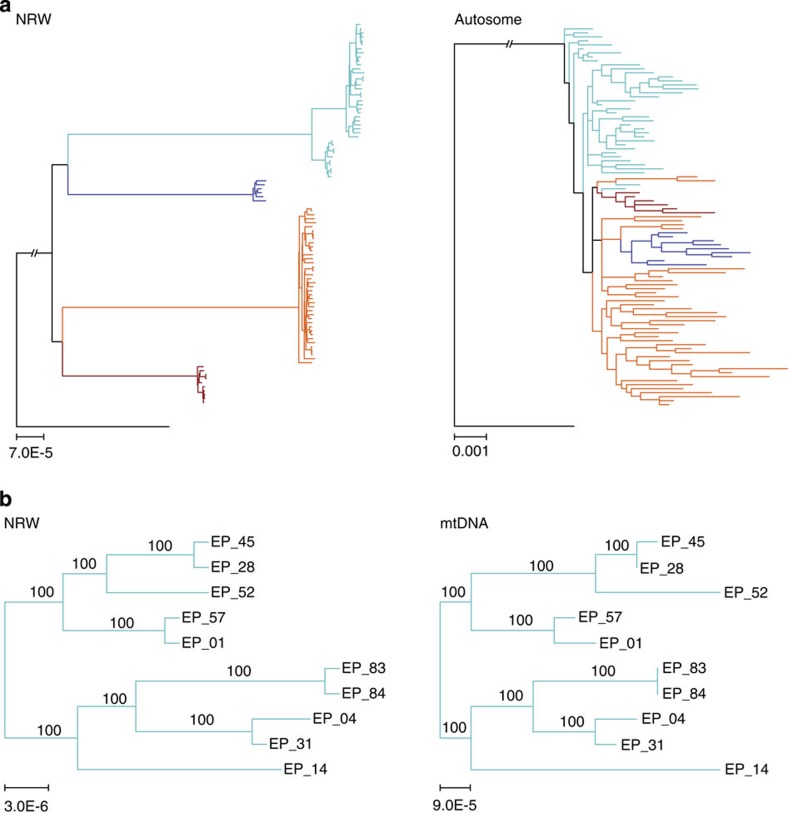
Phylogenetic relationships among flycatcher NRW haplotypes. (**a**) Monophyletic clustering of NRW haplotypes of each flycatcher species (left) but not of a 10 kb autosomal region (right). (**b**) Maximum likelihood trees representing the NRW (left) and mtDNA (right) gene genealogies of 10 Spanish pied flycatchers. Bootstrap percentages for maximum likelihood trees (100 replicates) are shown above branches. Colour codes: pied flycatcher, turquoise; Atlas flycatcher blue; collared flycatcher, red; semi-collared flycatcher, orange.

Estimates of population differentiation within and between flycatcher species revealed dramatic differences between the W chromosome and the rest of the genome. The black-and-white *Ficedula* flycatchers diverged 0.5–1.0 myr ago and still have a high proportion of shared autosomal polymorphisms and a moderate genome-wide *F*_*st*_ of 0.274–0.398. In contrast, NRW sequences were nearly fully sorted, with an *F*_*st*_ of 0.957–0.998 (Fig. 3b). This is consistent with an elevated rate of lineage sorting when *N*_e_ is low, with the Z chromosome (*F*_*st*_=0.435–0.531) being intermediate to autosomes and the W chromosome in this respect. Moreover, while genome-wide differentiation among populations within collared flycatchers and pied flycatchers are in the range of *F*_*st*_=0.012–0.064, within-species *F*_*st*_ for NRW sequences reached 0.186 (collared flycatcher) and 0.717 (pied flycatcher), respectively. The enhanced rate of lineage sorting is clearly evident from species-wise monophyletic clustering of NRW but not of autosomal sequences (Fig. 4a). It includes rapid fixation of derived synonymous as well as non-synonymous substitutions in coding sequences in each of the four flycatcher lineages (Supplementary Table 7).

### Co-segregation of W chromosome and mitochondrial DNA

Although Y chromosomes are evolutionarily uncoupled from mitochondrial DNA (mtDNA), maternal co-inheritance of both the W chromosome and mtDNA may introduce strong cyto-nuclear associations^39^,^40^ and affect sequence evolution of both these chromosomes in a similar fashion. However, phenomena such as paternal leakage of mtDNA or recombination in either molecule^41^ would weaken such associations. The availability of population genetic data for the W chromosome allows us, for the first time, to evaluate the strength of association between the NRW and mtDNA. In the absence of recombination and paternal leakage of mtDNA, we expect gene genealogies of individuals within a population to be identical between the two markers. To test this, we assembled the mitochondrial genomes of 10 pied flycatcher females and reconstructed maximum-likelihood (ML) cladograms for these individuals from both mtDNA and NRW sequences. Both markers yielded a fully resolved tree with identical topology, in line with the supposed co-inheritance/complete association of these two female-specific markers (Fig. 4b). Although it is difficult to quantitatively assess what rates of paternal leakage or recombination of mtDNA (or of NRW) can be excluded from the observed concordance between mtDNA and NRW trees, our results demonstrate evolutionary stability of the co-inheritance of these two chromosomes in an avian system.

## Discussion

A comprehensive assembly of the non-recombining region of the flycatcher W chromosome reveals a gene catalogue that primarily seems shaped by selection for maintaining ancestral expression levels of broadly expressed, dosage-sensitive sex-linked genes. This may be a common feature of the sex-limited chromosome in organisms with male and female heterogamety, inherently associated with needs following from a sex-determining system based on differentiated sex chromosomes. However, while Y chromosomes are also characterized by the presence of male reproductive genes and may constitute a battle ground for sexual selection^11^, we found no evidence for a corresponding enrichment of genes involved in female reproduction on the W chromosome. This would lead to a view of the W chromosome mainly representing a sex-linked appendix, although we cannot exclude that individual W-linked genes can evolve female-specific function in flycatcher or other bird species.

In the absence of recombination, Hill-Robertson interference should decrease the local *N*_e_, and thereby diversity, due to the effects of linked selection. Reduced nucleotide diversity is a hallmark of Y chromosomes^37^ (and other non-recombining chromosomes) and the severe reduction in polymorphism levels observed for the W chromosome of several flycatcher species seems somewhat extreme in this respect. Birds have significantly lower mtDNA diversity than mammals, which cannot be explained by differences in mutation rate or species *N*_e_ (ref. 40). Complete linkage disequilibrium between mtDNA and the W chromosome, for which we find support in a phylogenetic analysis, should reinforce HRI that is likely to be strong already within each of these chromosomes in female heterogametic organisms. Strong constraints on mtDNA genes for maintaining basic respiratory functions may therefore entail pervasive effects of background selection on diversity levels of the W chromosome^42^.

## Methods

### Assembly strategy

In theory, genomic reads from DNA that fail to map to a male-derived reference genome should correspond to sequences from the NRW. However, reference genomes are rarely complete and might particularly lack repetitive regions, likely yielding a considerable amount of ‘false positives’ in such an approach. Moreover, contamination in sequenced samples as well as reads containing sequencing errors would appear as W-chromosomal. At the same time, ‘false negatives’ could arise by reads from the W chromosome mapping to paralogous sequences in the reference genome. Our initial observation of similar proportions of unmapped reads to the male reference genome in male (mean 3.09%, 95% confidence interval 1.40–4.78%) and female (mean 3.61%, 95% confidence interval 1.57–5.65%) re-sequencing confirmed that this was not a viable strategy for enrichment of W chromosome sequences.

As an alternative strategy, we made a *de novo* genome assembly from sequencing of female DNA. Genomic re-sequencing reads from 40 collared flycatcher females with an average coverage of 15.1 X were generated with Illumina paired-end sequencing technology on a HiSeq 2000 instrument, with 450 bp insert libraries sequenced from both ends using 100 cycles (sequence data available in the European Nucleotide Archive, ENA, accession number PRJEB7359). Sequences were filtered for PCR duplications and trimmed for base quality with CONDETRI^43^ and Illumina adapter sequences with CUTADAPT^44^. We first used trimmed reads (excluding reads mapping to mitochondrial DNA) from one of the individuals with highest coverage (H_354_F; 23.5 X coverage) and assembled them into contigs with SOAPDENOVO2^45^ using a k-mer value of 23. Data from all 40 females were then used for merging the contigs into scaffolds based on paired-end information. Finally, data from 10 females were used for gapclosing with SOAPGAPCLOSER^45^. The assembly was repeat masked (see below) and consisted of both scaffolds (merged contigs) and singletons (contigs that could not be merged into scaffolds); we collectively refer to both categories as ‘scaffolds’ for the sake of simplicity. The use of short insert size (450 bp) libraries in the generation of re-sequencing data coupled with a high repeat density (Supplementary Table 2) rendered contigs and scaffolds relatively short (Supplementary Table 1).

When separately mapping re-sequencing reads from females and males onto a female assembly, scaffolds originating from the W chromosome should in principle be covered by female reads only, while having zero male coverage. However, in practice, ambiguities may arise in repeat-rich regions or due to the mapping of reads with imperfect matches, and such a ‘black-and-white’ pattern is unlikely to be seen. We therefore used an adapted version of the chromosome quotient (CQ) method^46^, which distinguishes sex-specific sequences (scaffolds) in an assembly based on relative coverage of male and female reads. Rather than using the number of mapped reads per scaffold to define the male:female (M:F) ratio threshold as done by Chen *et al*.^46^, we more stringently calculated the M:F ratio for each scaffold from the median per-site coverage of trimmed reads mapping with zero mismatches. This means that reads with real variation (for example, SNPs) are lost along with reads containing sequencing errors, but it will vastly decrease the incidence of false positives. We used pooled sequence data from 10 females and 10 males, respectively, which summed up to a mean genome-wide coverage of 65.1 (females) and 63.3 X (males) after removing reads with mismatches; recall that these values are for mostly diploid chromosomes, such that the haploid W chromosome should be expected to have half the genome-wide female coverage. The M:F quota threshold was set to 0 (meaning that median male coverage had to be 0), while female median coverage had to be at least 15 X, for accepting a scaffold as W-linked. We consider these criteria to be highly stringent and potentially implying that some scaffolds from the W chromosome would remain undetected, however, the benefit of effectively excluding false positives was given priority. Using this method, we found 1,920 NRW scaffolds with a total length of 6.9 Mb. As a comparison, we used the CQ method with all trimmed reads and default settings (script downloaded from http://tu08.fralin.vt.edu/software/CQcalculate) and retrieved 1,398 scaffolds out of which 1,383 were already found by the modified median method.

The assembly of NRW scaffolds was improved by merging any overlapping sequence using cap3 ref. 47). As a further step of improvement, we used L_RNA_SCAFFOLDER^48^ and BESST_RNA ( https://github.com/ksahlin/BESST_RNA) to scaffold sequences using RNA transcripts and RNA-seq reads, respectively. We used collared flycatcher RNA-seq data from four females and seven tissues^22^ available in Short Read Archive, SRA (accession numbers: ERX144598, ERX144614-16, ERX144618, ERX144642-44, ERX144646, ERX144666-68, ERX144670, ERX144672-74, ERX144690, ERX144691-94, ERX144696, ERX144729, ERX144731) and assembled the reads into transcripts (for L_RNA_SCAFFOLDER) with TRINITY^49^. This only merged a small fraction of the NRW scaffolds, reducing the total number from 1,920 to 1,884.

### Repeat annotation and repeat landscape analyses

We updated the flycatcher repeat annotation by *de novo* screening the FicAlb1.5 assembly^32^ for flycatcher-specific repeats. This was done by using REPEATMODELER (version 1.0.5; http://www.repeatmasker.org/RepeatModeler.html), a repeat identification and modelling package consisting of RECON (version 1.07), REPEATSCOUT (version 1.0.5) and TANDEM REPEATS FINDER (version 4.0.4) using RMBLAST ( http://www.repeatmasker.org/RMBlast.html). The resultant repeat candidate library was manually curated following Lavoie *et al*.^50^. BLASTN searches of long terminal repeat retrotransposon-like repeat candidates were conducted against FicAlb1.5 and up to 50 of the best hits were extracted along with 1 kb of flanking sequence, respectively. Subsequently, the consensus sequence of each candidate was aligned with its BLAST hits using MAFFT (version 6; ref. 51). For each of these alignments, we generated a manually inspected consensus sequence that was termed ‘complete’ if it spanned a region in the alignment flanked by unique, single-copy sequence. We combined our flycatcher repeat library with previously known avian repeat elements (mainly from chicken and zebra finch) available in REPBASE ( http://www.girinst.org/repbase/index.html) into a custom repeat library for annotation and masking both the FicAlb1.5 genome assembly and the new NRW assembly using REPEATMASKER (v3.2.9; http://www.repeatmasker.org/RMDownload.html).

We calculated pairwise distances of repeat elements from their respective repeat consensus sequences using the calcDivergenceFromAlign.pl script from the REPEATMASKER program package. Hyper-mutable CpG sites were removed during the calculation under the Kimura 2-parameter model^52^ and the ‘.align’ REPEATMASKER output file was converted into a table file^53^. We then plotted repeat landscapes by estimating the cumulative amount of masked bp per repeat group divided by the size of the respective chromosomal class (Supplementary Fig. 2).

### Gene annotation

We used MAKER (v 2.31; ref. 54) for gene prediction with several types of evidence as input: (i) ENSEMBL (release 77) annotated protein sequences from chicken, zebra finch (*Taeniopygia guttata*), anole (*Anolis carolinensis*) and Chinese softshell turtle (*Pelodiscus sinensis*), as well as the CEGMA core protein data set, (ii) RNA-seq data from phylogenetically diverse bird species^55^, (iii) flycatcher transcript predictions for version FicAlb1.5 of the flycatcher genome (that is, not including the NRW) obtained by CUFFLINKS v2.1.1, (iv) flycatcher-specific repeat libraries (see above) and (v) flycatcher-specific Augustus training parameters. Transcripts from MAKER were BLASTed against all flycatcher Z chromosome genes downloaded from ENSEMBL. Fifty-eight transcripts hit a total 40 different genes, which means there were multiple transcripts for several of the genes. Manual inspection showed that this was a combined result of short scaffolds in the assembly and the fact that MAKER cannot predict genes spanning several scaffolds. It may therefore output several separate predictions for one gene. To find potentially missing regions between the predicted transcripts, we aligned the 40 Z chromosome genes back to the NRW assembly and checked that they overlapped with the predicted regions. If a gene spanned more than one scaffold, the scaffold sequences were concatenated with an arbitrary gap size of 500 Ns in between. The same procedure was repeated from the step of mapping MAKER transcripts, but in this case mapping to all chicken Z chromosome genes from ENSEMBL and to known genes from the chicken W chromosome. As a complementary approach, we also used CUFFLINKS (v2.2.1; ref. 56) to improve transcript annotation. This approach identified 11 additional NRW genes. After merging scaffolds containing exons from the same gene, the number of scaffolds decreased from 1,884 to 1,779.

All genes were manually inspected in IGV^57^ to test whether there were reads spanning the exon junctions. Five genes had internal stop codons and/or frame shifts and are likely pseudogenes (*IFNB*, *FCHO2*, *SMC2, NTRK2*, *HOMER1*). Another three genes lacked support from RNA-seq, that is, were not detected as expressed. Two of these were novel genes (homologues of ENSGALG00000025865 and ENSFALG00000014210) with relatively low alignment score and were excluded from further analysis. The third gene (*RNF38*) had a highly supported BLAST hit and was included.

Predicted transcripts from MAKER and CUFFLINKS that lacked hits on the flycatcher Z chromosome, chicken Z chromosome or chicken W chromosome were BLASTed against all available proteins in the NR database at NCBI. Two transcripts from a single NRW scaffold hit one gene each (*SMAD4*, *MEX3C*) in several phylogenetically diverse bird genomes (including ingroups to both chicken and flycatcher) with high confidence and had high RNA-seq support in our data. We consider it unlikely that both genes have been independently deleted in chicken and flycatcher, and that at least an as plausible explanation is that they had failed to be included in the respective genome assembly. Moreover, since *SMAD4* and *MEX3C* are located close to each other in a region of human chromosome 18 that is homologous to the avian Z chromosome, we assume that they are located on the flycatcher Z chromosome; their chromosomal location in other bird species is not known since only a limited number of avian genomes have scaffolds assigned to chromosomes.

### Quantification of gene expression

TOPHAT (v2.0.12; ref. 56) and CUFFLINKS were used to map RNA-seq data to final NRW gene annotations and estimate transcript abundance. CUFFLINKS-derived FPKM values were extracted using in-house scripts and further normalized to zFPKM using the approach by Hart *et al*.^58^. Statistical analyses of gene expression patterns were performed using R v3.1.1. Expression breadth (*τ*) was estimated according to Yanini *et al*.^59^. *P* values were Benjamini–Hochberg-corrected for multiple testing in all cases involving gene expression data.

### Phylogenetic analysis of gametologous genes

Evolutionary strata on sex chromosomes are understood as more or less discrete events of cessation of recombination between sex chromosomes, with inversions on the sex-limited chromosome being a likely cause to the arrest of recombination^9^. To test for the presence of evolutionary strata, orthologous and, if available, gametologous sequences from collared flycatcher, chicken, zebra finch, ostrich (*Struthio camelus*) or anole were aligned using PRANK^60^. ML trees using ostrich or anole as outgroup were generated using GARLI (v0.96beta8; ref. 61). The programme was run 50 times using a two-rates (transition and transversion) nucleotide model, setting observed values as equilibrium-state frequencies and using four discrete gamma-distributed rate categories. We then performed a 500 replicate bootstrap analysis. We summarized and mapped the bootstrap values to branches of the best ML tree using SUMTREES v3.3.1 of the DENDROPY package v3.12.0 (ref. 62). Genes were classified as belonging to an ‘old’ evolutionary stratum that was established before the split of flycatcher and chicken lineages if the Z-linked gametolog of flycatcher and chicken clustered together, with the W-linked flycatcher gametolog being sister to those lineages. Genes with the flycatcher Z-linked and W-linked gametologs clustering, with chicken Z-linked gametolog being sister, were classified as belonging to a ‘young’ evolutionary stratum that was established after the split of flycatcher and chicken lineages. A bootstrap support value for either of the above topologies of 0.70 was requested for inference of stratum affiliation. For the purpose of this study, we do not further examine the possible presence of additional strata.

Patterns of sex chromosome evolution are usually characterized by the observation of progressively younger evolutionary strata towards the PAR. The pattern seen for the flycatcher Z chromosome was no exception, with the young stratum represented by genes at positions 1.4–27.1 Mb and the old stratum by genes at positions 28.6–68.6 Mb; the minute PAR is located in the very beginning of the flycatcher Z chromosome^21^. To test for non-random survival of genes in the young stratum in the parallel flycatcher and chicken lineages, we noted the number of shared genes on the flycatcher and chicken Z chromosome in the segment ≤27.1 Mb, and the number of surviving genes on the NRW of flycatcher and chicken, respectively. A chi-square test was used to statistically test if the number of genes common to the NRW of the two species was higher than expected by chance.

Two genes did not conform to the discrete distribution of genes along the flycatcher Z chromosome with respect to inferred cessation of recombination either before or after the split of flycatcher and chicken lineages. Specifically, *CTIF* at 1.5 Mb and *KIF2A* at 20.0 Mb were classified as belonging to the old stratum despite being located within the segment <27.1 Mb. There are several possible explanations to this, including mis-assembly of these genes in the reference genome. This should be further investigated when additional avian genome assemblies become available, however, we do not consider it having a major effect on the definition of evolutionary strata in this study.

### Substitution rate estimation

We used a codon model in PAML package (v4.7; ref. 63) to estimate synonymous (*d*_S_) and non-synonymous (*d*_N_) substitution rates per branch per gene using the GARLI ML gene tree topologies. PAML was also used for estimating pairwise *d*_S_ between gametologous flycatcher gene pairs. We excluded genes for which *d*_S_>1.5. For calculating *d*_S_ and *d*_N_ of Z-linked and W-linked gametologs since sex chromosome divergence, we summed the CODEML output estimates of the branches leading to the flycatcher Z (W) chromosome gametolog since its split from the W (Z) chromosome. For genes that ceased to recombine subsequent to flycatcher-chicken divergence (that is, the split between Passeriformes and Galliformes), this included the terminal flycatcher branch and the internal branch leading to flycatcher and zebra finch. For genes that ceased to recombine before flycatcher–chicken divergence, this also included the internal branch leading to flycatcher, zebra finch and chicken. For estimates of omega (*d*_N_/*d*_S_) we allowed for three different values: one for the branch leading to the flycatcher Z-linked gametolog since the split from the W chromosome lineage, one for the branch leading to the flycatcher W-linked gametolog since the split from the Z chromosome lineage, and one for the rest of the tree.

The male mutation bias (*α*) was estimated from the relationship between synonymous substitution rates of gametologous genes on the Z chromosome and on the NRW as *d*_S_ (Z)/*d*_S_ (NRW)=2/3*α*+1/3, that is, with the female mutation rate set to 1. This simple formula is derived from the fact that the Z chromosome is transmitted two-thirds of the time through the male germ line and one-third of the time through the female germ line.

### Variation calling

Base quality re-calibrated female re-sequencing reads (mean coverage=15.2 X) of 40 collared flycatchers, 39 pied flycatchers (*Ficedula hypoleuca*), six Atlas flycatchers (*F. speculigera*), 10 semi-collared flycatchers (*F. semitorquata*) and one red-breasted flycatcher (*F. parva*; ENA accession number PRJEB7359) were mapped to the full FicAlb1.5 reference genome together with the NRW assembly (all sequences soft masked). We extracted reads exclusively mapping onto the NRW sequences and used these for haploid SNP calling per population with GATK, v3.2.2 (ref. 64). Because of the lack of known SNPs for the NRW and a relatively short reference sequence, we could not use the recommended VARIANTRECALIBRATOR for filtering SNPs. Instead we used hard filtering as suggested by GATK’s Best Practice ( https://www.broadinstitute.org/gatk/guide/best-practices). To get a stringent set of SNPs and decrease the risk of including false positives, we, in addition, applied a coverage filter by defining the expected coverage to half of the mean coverage for autosomal scaffolds per individual and masking sites in that individual if the coverage was lower than half or higher than twice the expected coverage. If less than seven individuals per population (six for Atlas flycatcher due to the lower sample size) remained after coverage filtering, the site was excluded entirely for that population. Since the W chromosome is haploid, it contains no heterozygous sites. However, collapsed regions in a NRW assembly can appear heterozygous if collapsed copies are slightly divergent. To identify such ambiguous sites, we also performed diploid SNP calling for each population and extracted all positions where more than one individual from a population was called as heterozygous after hard filtering. These positions (6,348) were then masked in our haploid SNP set for all individuals.

As a further validation step, we used re-sequencing data from 103 male flycatchers of all species (PRJEB7359) and called SNPs in the same manner as above. In total, eight short scaffolds had male SNPs. Manual inspection of mapped data from both females and males showed that one of the sequences was a chimera and that the others were not of W chromosome origin. The chimeric scaffold was pruned to remove the incorrect part, while the other seven scaffolds were removed completely; the W chromosome assembly was in this way reduced by 15.6 kb and the number of scaffolds to 1,772.

### Population genomic analyses

Genetic differentiation (*F*_*ST*_) of NRW sequences between species and populations was estimated using a hierarchical estimation procedure implemented in the HIERFSTAT package in R ( http://www2.unil.ch/popgen/softwares/hierfstat.htm). Genetic diversity of the NRW for each species was estimated as the mean number of pairwise differences per site (*π*) using custom R scripts.

### Mitogenome assembly

Mitochondrial genomes (mtDNA) for 10 female pied flycatcher individuals from Spain were assembled by mapping reads to the published mitochondrial genome of the collared flycatcher (GenBank accession number: KF293721) using MITOBIM^65^. In MITOBIM, the mapping assembly consists of several steps. In the first step, MIRA v3.4.1.1 is used to generate anchor contigs from reads that map to highly conserved regions of the reference sequence. In the next step, reads that overlap with either side of the anchor contigs are mapped, thereby extending the anchor contigs and reducing the gaps between them. This process (‘*in silico* baiting’) is iterated until the programme converges on a final sequence. The sequences obtained from MITOBIM were aligned against the reference genome using MUSCLE v3.8 with default settings^66^. Visual inspection of the sequences in SEAVIEW v. 4 (ref. 67) revealed that MITOBIM was unable to unambiguously reconstruct the control region in pied flycatchers. The control region was therefore removed for the phylogenetic analysis of these samples.

### Phylogenetic analyses of haplotypes

For phylogenetic analyses of W chromosome data, the NRW sequences of all 96 individuals were extracted from VCF-files, converted to fasta format and concatenated. All filtered sites (see above) were re-coded as missing data and positions that were coded as missing data in all 96 individuals were removed. The concatenated and filtered NRW sequences had an alignment length of 3,183,488 nucleotide positions. To obtain a tree representative of nuclear DNA, we randomly selected 10 kb of continuous autosomal sequence from the FicAlb1.5 reference genome^32^. We excluded regions closer than 20 kb from any exonic region based on the Ensembl gene annotation of the collared flycatcher reference genome version FicAlb1.4, as well as regions with >20% sites hard-masked by REPEATMASKER. We phased the selected region with FASTPHASE v1.4.0 (ref. 68), using sequence data from 198 flycatcher individuals. To minimize phasing errors, we recoded all heterozygous genotypes with <80% posterior phasing probability as missing data and then randomly chose one haploid sequence from each of the same 96 individuals as in the NRW data set.

We constructed ML gene trees from both data sets using RAXML v 8.0.2 (ref. 69) under the GTRGAMMA evolutionary model (the general time reversible model with C-distributed rate variation among sites) using *F. parva* as an outgroup. Using the same settings, we also inferred the ML gene genealogy from the mitochondrial genomes of 10 Spanish pied flycatcher individuals. The topology of the resulting mtDNA gene tree was then compared with the corresponding subclade of the NRW-based tree.

## Additional information

**Accession codes**: Sequence data have been deposited in the European Nucleotide Archive under the BioProject accession code PRJEB7359. Assembled reads are available under codes CVIS01000001 to CVIS01001807, within BioProject PRJEB7359.

**How to cite this article:** Smeds, L. *et al*. Evolutionary analysis of the female-specific avian W chromosome. *Nat. Commun.* 6:7330 doi: 10.1038/ncomms8330 (2015).

## Supplementary Material

Supplementary Information Supplementary Figures 1-6, Supplementary Tables 1-7 and Supplementary References

## Figures and Tables

**Table 1:** Identified genes on the non-recombining part of the collared flycatcher W chromosome.

**Locus**	**Gene ID of Z-linked gametolog**	**Position on Z (bp)**
*SMAD7W*	ENSFALG00000008945	1397439
*CTIFW*	ENSFALG00000008943	1499112
*SMAD2W*	ENSFALG00000008934	1861091
*C18orf25W*	ENSFALG00000008886	2674578
*ATP5A1W*	ENSFALG00000008875	2739445
*UBAP2W*	ENSFALG00000009935	8306662
*DCAF12W*	ENSFALG00000009894	8430857
*UBAP1W*	ENSFALG00000009887	8471437
*FAM219AW*	ENSFALG00000009864	8589404
*VCPW*	ENSFALG00000009780	9497605
*GOLPH3W*	ENSFALG00000002160	10866720
*ZFRW*	ENSFALG00000002149	10975563
*SUB1W*	ENSFALG00000002143	11012552
*NIPBLW*	ENSFALG00000002058	12582107
*PRKAA1W*	ENSFALG00000002013	14086131
*RPL37W*	ENSFALG00000002006	14105142
*ZNF131W*	ENSFALG00000011128	14898488
*SNX18W*	ENSFALG00000010790	17817091
*MIER3W*	ENSFALG00000010987	18857934
*ZSWIM6W*	ENSFALG00000011056	20574573
*KIF2AW*	ENSFALG00000011073	20905811
*SREK1W*	ENSFALG00000009835	22411155
*MRPS36W*	ENSFALG00000009866	23541133
*COL4ABPW*	ENSFALG00000010073	25692644
*TNPO1W*	ENSFALG00000010292	26868143
*MAP1BW*	ENSFALG00000010312	27144807
*RFX3W*	ENSFALG00000010515	28550597
*CDC37L1W*	ENSFALG00000010536	29009066
*CHD1W*	ENSFALG00000010499	30131651*
*RASA1W*	ENSFALG00000010471	30131651*
*GNAQW*	ENSFALG00000003294	36520702
*novel2*	ENSFALG00000006359	36685973*
*HNRNPKW*	ENSFALG00000012478	36685973*
*SPINW*	ENSFALG00000012406	40533877
*NFIL3W*	ENSFALG00000014613	41771706
*HINT1W*	ENSFALG00000004845	42292467
*KCMF1W*	ENSFALG00000005137	44466461
*RNF38W*	ENSFALG00000000978	46376861
*FEM1CW*	ENSFALG00000002587	53787080
*ZFAND5W*	ENSFALG00000010733	57062641
*ZNF462W*	ENSFALG00000002876	58249660
*ARRDC3W*	ENSFALG00000009068	64237725
*CKMT2W*	ENSFALG00000012745	68100720
*novel1*	ENSFALG00000014649	68591987

Following convention, a ‘*W*’ has been added to each gene symbol to denote that it refers to a W-linked gametolog.

^*^The location of these genes is approximated since the corresponding scaffolds have not been ordered by confidence.
