# Supplementary material for: Evolutionary analysis of the female-specific avian W chromosome
Source: Nat Commun. 2015 Jun 4;6:7330. doi: 10.1038/ncomms8330 (PMC4468903; doi:10.1038/ncomms8330)
Supplement: Supplementary Information — Supplementary Figures 1-6, Supplementary Tables 1-7 and Supplementary References [file ncomms8330-s1.pdf]

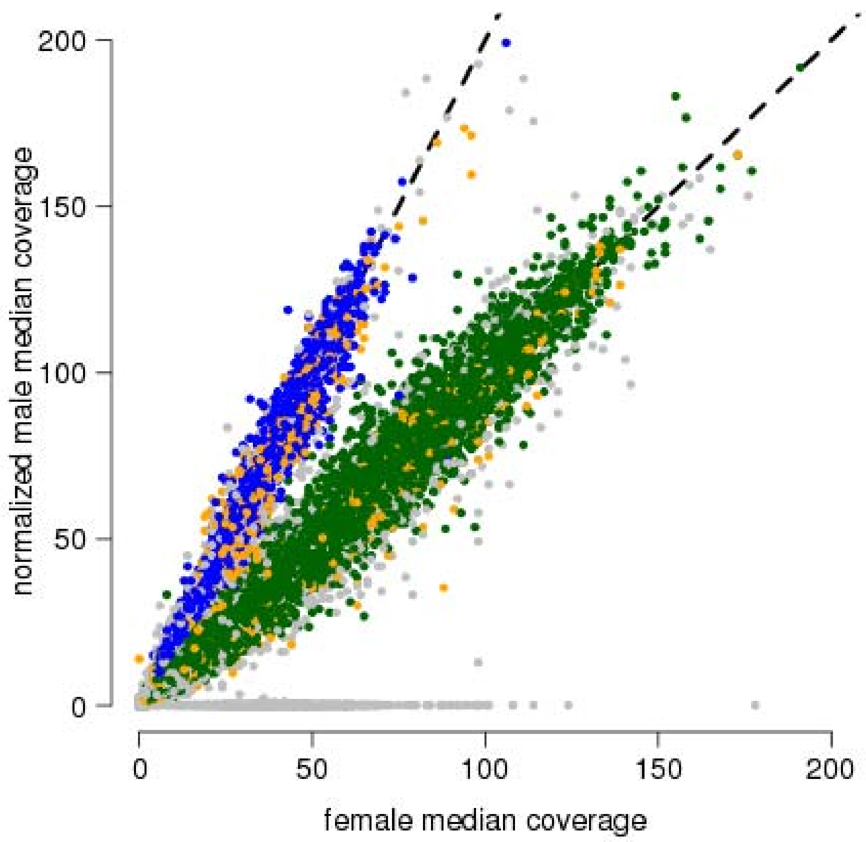

Supplementary Figure 1 | Correlation between male and female re-sequencing coverage to the female-derived genome assembly. Re-sequenced reads were mapped to the female assembly with BWA (v0.7.4)[1]. Each dot represents the median coverage per scaffold in the assembly, with chromosomal location inferred from FicAlb1.5 male reference genome (scaffolds aligned with LASTZ[2]). Male coverage was normalized by female coverage, to make the results from the two sexes comparable. Dashed lines refer to expected 2:1 and 1:1 relationships for Z chromosome and autosomal sequences, respectively. Colour codes: blue, Z chromosome; green, autosomes; yellow, unassigned (unknown chromosomal location); grey, scaffold sequence not present in the male reference genome.

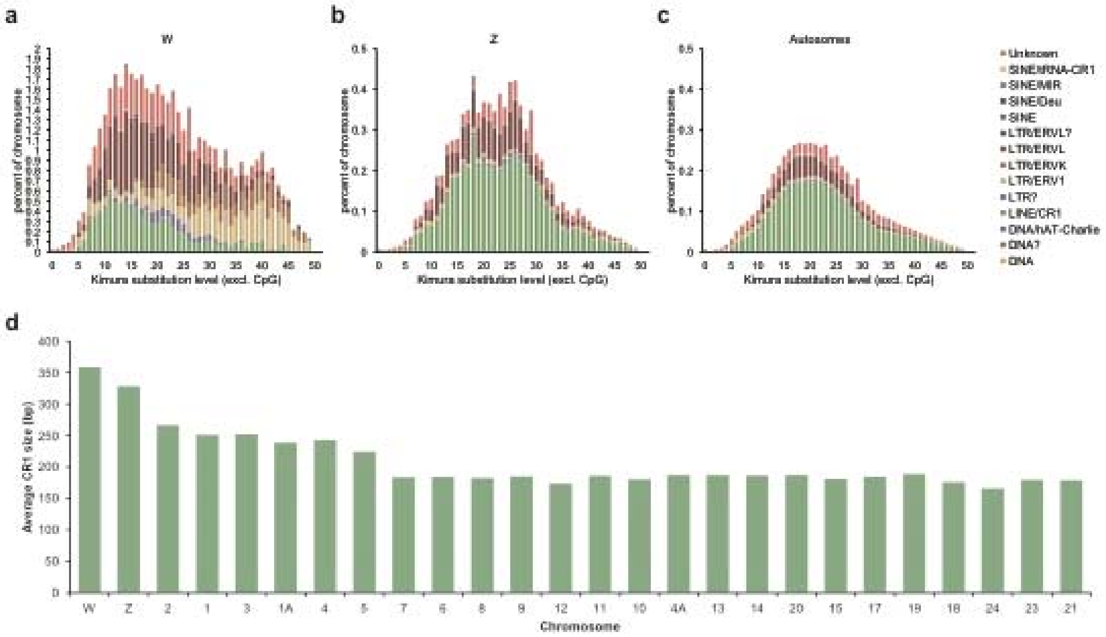

Supplementary Figure 2 | Transposable elements and the different chromosomal classes in the collared flycatcher genome. a) TE divergence landscapes on the non-recombining region of the W chromosome, **b)** the Z chromosome, and **c)** autosomes. Bins of repeat substitution level are based on the pairwise distance of repeat element sequences to their respective consensus sequences. Repeat types are named according to the annotation in RepeatMasker. **d**) Average copy size of chicken repeat 1 (CR1) elements on NRW, Z chromosome, and autosomes. Autosomes are arranged in descending order of chromosome size.

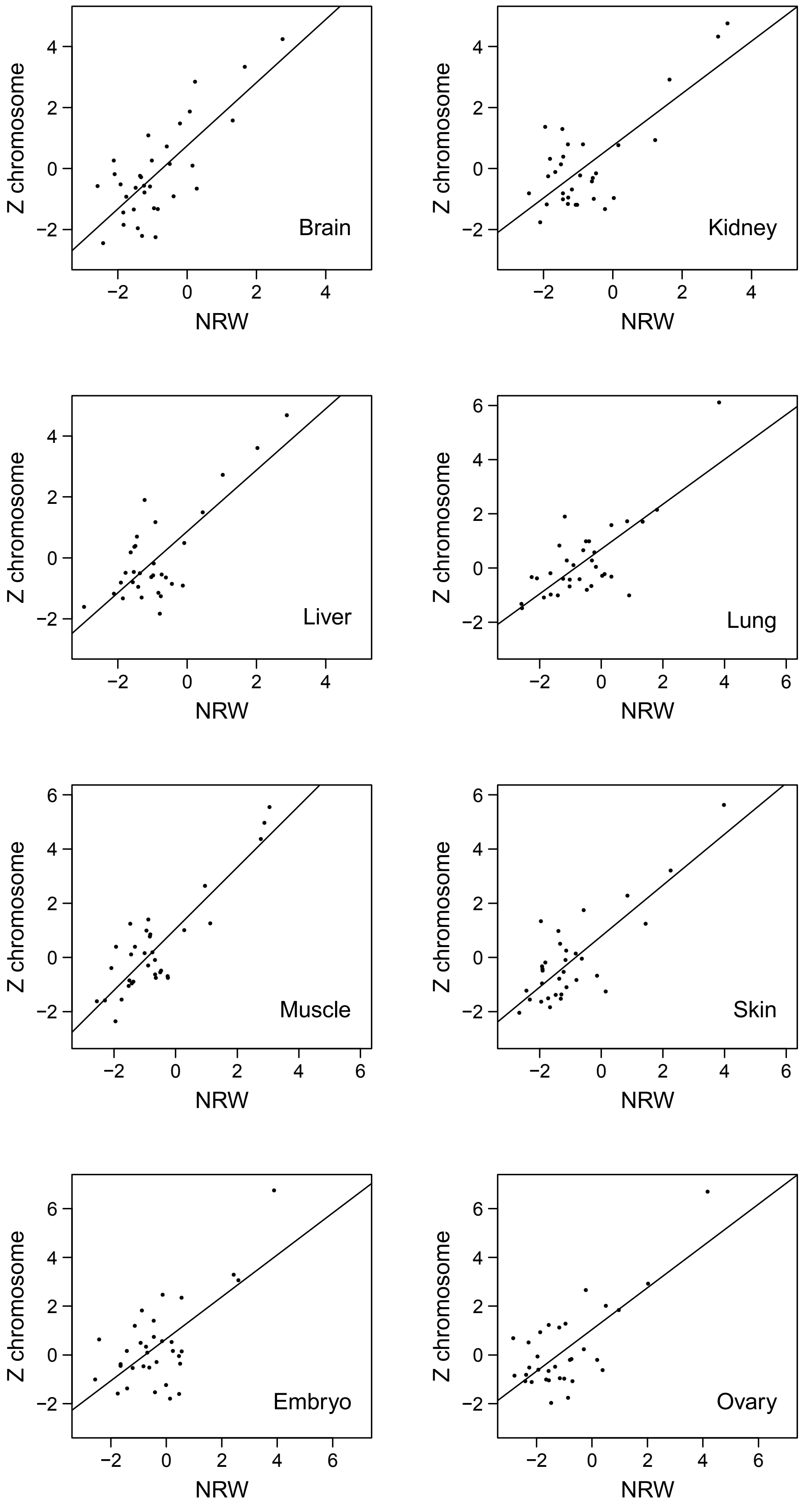

Z chromosome
NRW
Brain
Kidney
Liver
Lung
Muscle
Skin
Embryo
Ovary

Supplementary Figure 3 | Correlation between female expression levels of the Z-linked and W-linked copy of each gametologous gene pair in different tissues. Expression levels are given as $\log_2$ zFPKM values.

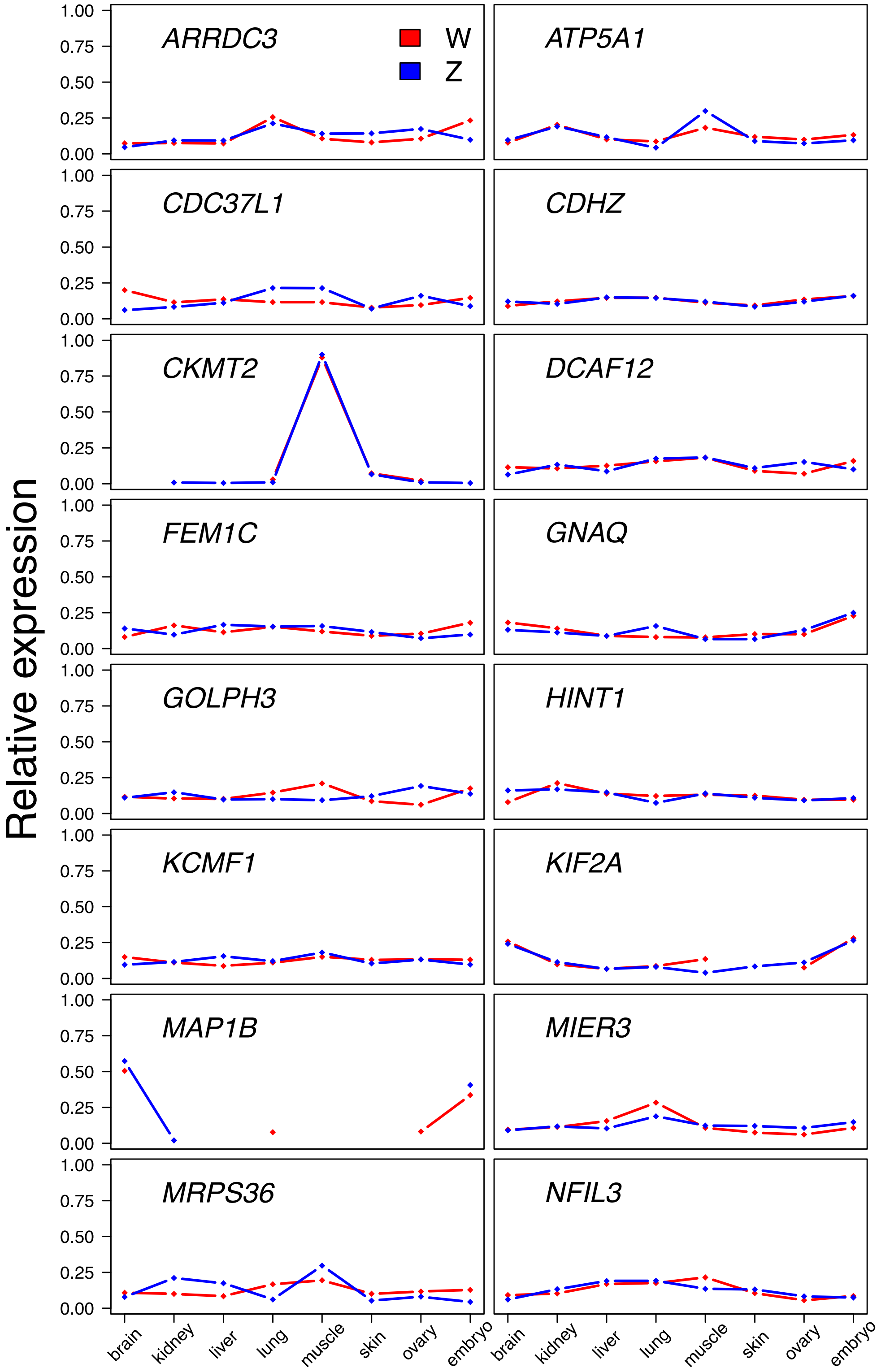

Relative expression
1.00
0.75
0.50
0.25
0.00
W
Z
ARRDC3
ATP5A1
CDC37L1
CDHZ
CKMT2
DCAF12
FEM1C
GNAQ
GOLPH3
HINT1
KCMF1
KIF2A
MAP1B
MIER3
MRPS36
NFIL3
brain
kidney
liver
lung
muscle
skin
ovary
embryo

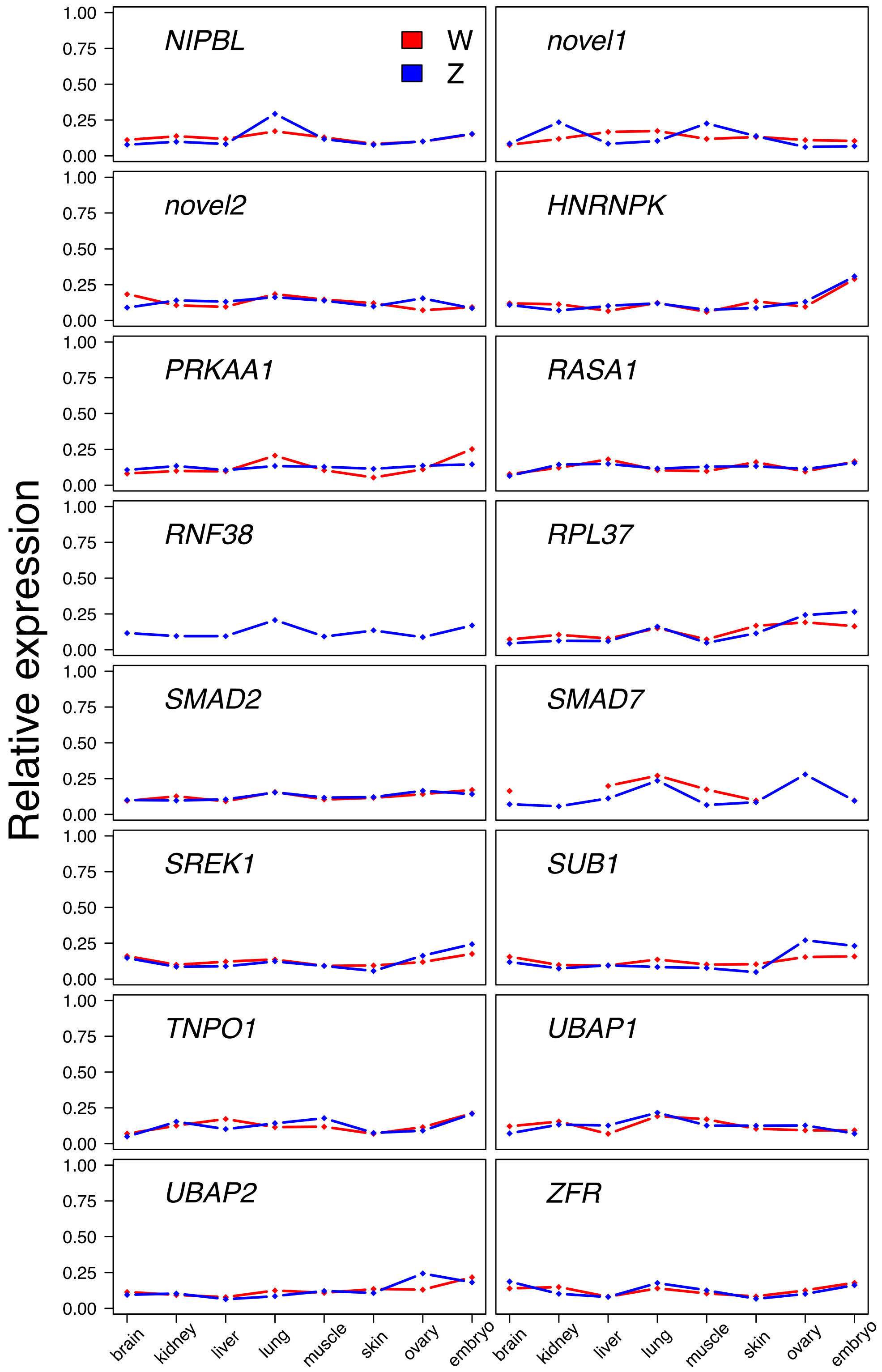

Relative expression
NIPBL
W
Z
novel1
novel2
HNRNPK
PRKAA1
RASA1
RNF38
RPL37
SMAD2
SMAD7
SREK1
SUB1
TNPO1
UBAP1
UBAP2
ZFR
1.00
0.75
0.50
0.25
0.00
brain
kidney
liver
lung
muscle
skin
ovary
embryo

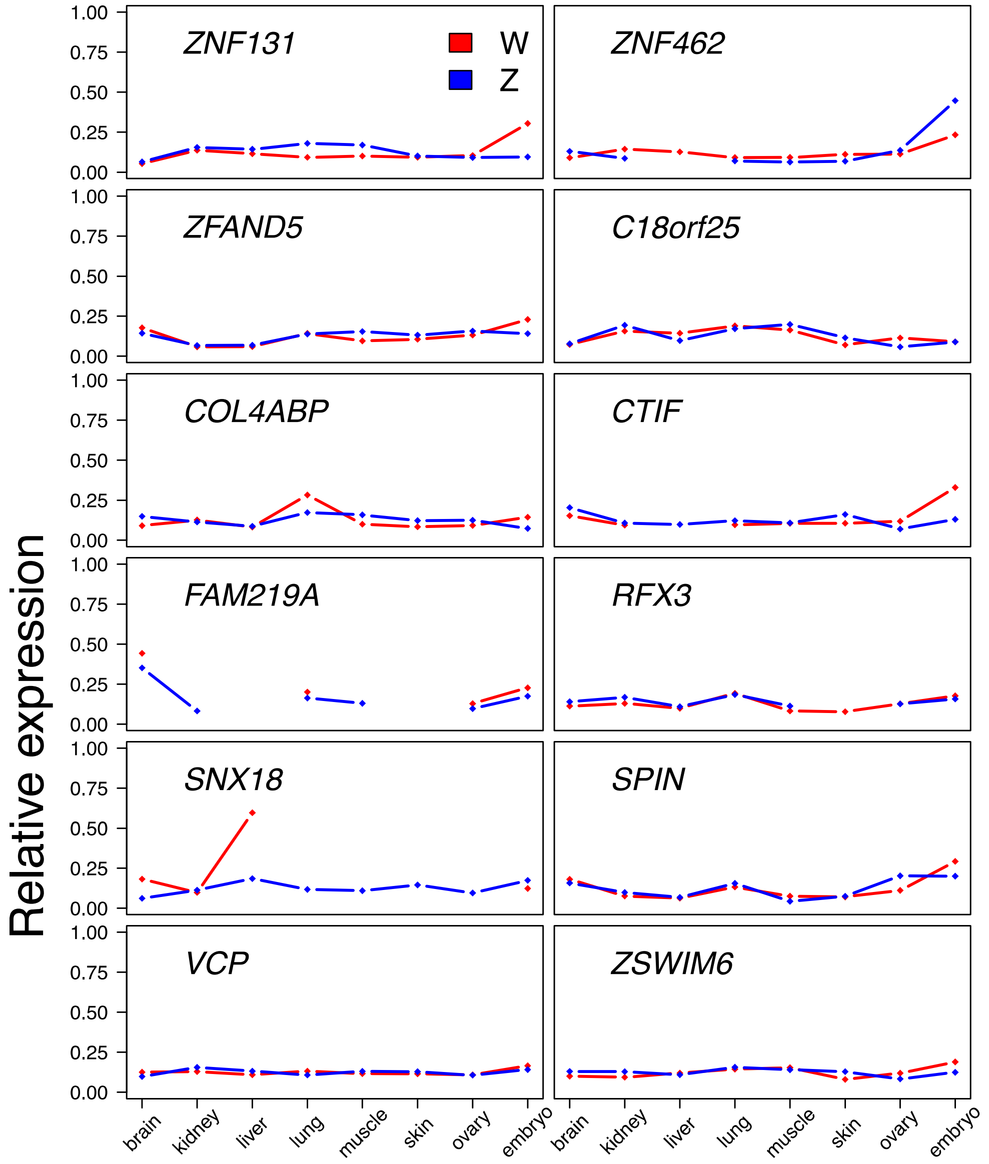

Supplementary Figure 4 | Expression profiles of Z and W gametologs across tissues.

Relative expression level in each of the eight tissues examined are shown.

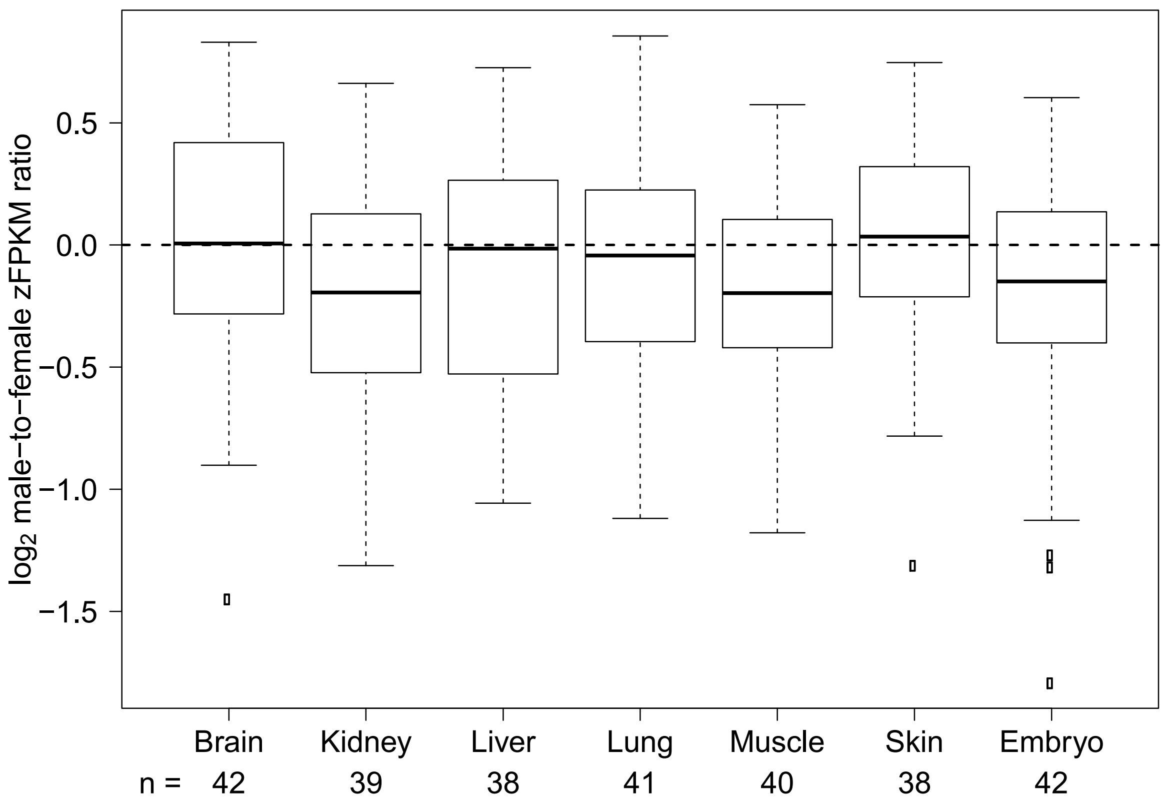

Supplementary Figure 5 | Male-to-female expression ratios ($\log_2$) for gametologous genes on the collared flycatcher sex chromosomes. Box plots for seven different tissues are shown. Boxes show distribution quartiles with the median in bold. Whiskers show minimum and maximum of the distribution unless this is more than 1.5 times the interquartile distance. Outliers exceed this limit.

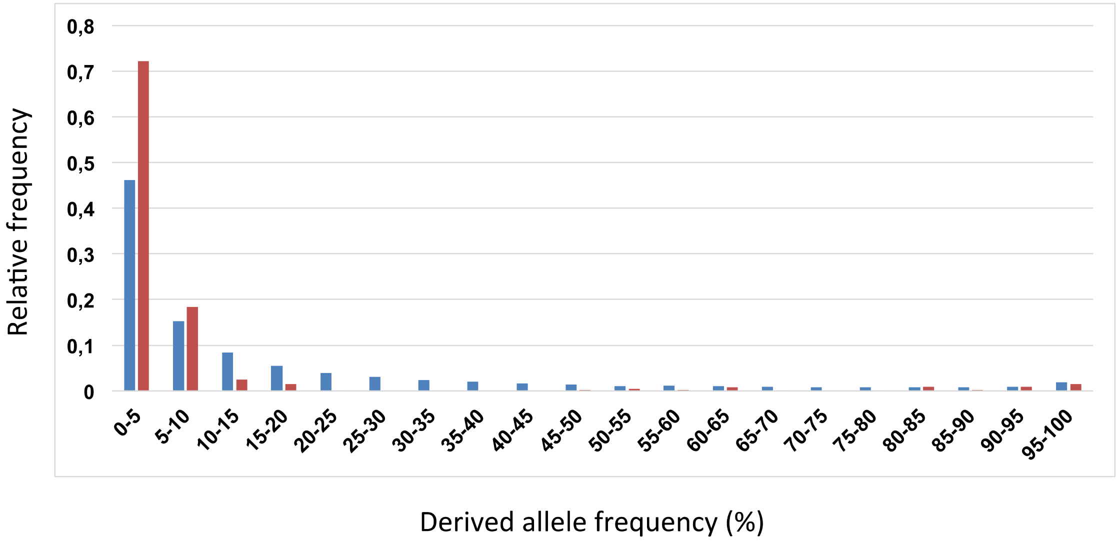

Supplementary Figure 6 | Unfolded site frequency spectra for collared flycatcher. Colour code: blue, autosomal sequences; red, NRW sequences.

Supplementary Table 1 | Summary statistics for the female collared flycatcher genome assembly

	Genome	W chromosome	Improved W[a]
# scaffolds	47,848	1,913	1,772
Total length (bp)	997,866,922	6,875,665	6,936,645
N50 (bp)	65,275	5,200	5,701
Mean length (bp)	20,855	3,590	3,915

[a] After scaffolding using RNA-seq information.

Supplementary Table 2 | Repeat density in different chromosomal classes in the collared flycatcher genome. Densities are expressed as the percentage of sequence derived from transposable elements.

Repeat type	W chromosome	Z chromosome	Autosomes
DNA	0,00	0,01	0,01
DNA?	0,00	0,01	0,02
DNA/hAT-Charlie	0,01	0,01	0,00
LINE/CR1	8,93	5,15	3,68
LTR?	1,67	0,14	0,07
LTR/ERV1	8,89	0,25	0,18
LTR/ERVK	5,37	0,15	0,06
LTR/ERVL	12,69	1,77	0,85
LTR/ERVL?	0,29	0,05	0,03
SINE	0,00	0,01	0,02
SINE/Deu	0,01	0,01	0,02
SINE/MIR	0,00	0,01	0,02
SINE/tRNA-CR1	0,02	0,02	0,02
Unclassified	10,61	1,17	0,94
Total	48,48	8,77	5,93

Supplementary Table 3 | Analysis of enrichment for gene ontology of collared flycatcher W chromosome genes

GO:ID	*p*-value	Corr. *p*-value	N_1	N_2	Definition	Ontology
GO:0005634	3.84e-05	0.093	20	124	nucleus	Cellular component
GO:0005667	2.69e-04	0.327	3	3	transcription factor complex	Cellular component
GO:0005515	4.09e-04	0.332	33	312	protein binding	Molecular function
GO:0043547	9.52e-04	0.580	3	4	positive regulation of GTPase activity	Biological process
GO:0044822	2.16e-03	0.883	8	35	poly(A) RNA binding	Molecular function
GO:0000790	2.78e-03	0.883	3	5	nuclear chromatin	Cellular component

N_1 Number of genes on NRW. N_2 Number of genes on the Z chromosome

Supplementary Table 4 | Median inter-individual expression variance in males of Z-linked genes with (i.e., gametologous genes) or without a retained W-copy.

Tissue	With W-linked gene copy	Without W-linked gene copy
Brain	0.047	0.125
Embryo	0.063	0.093
Kidney	0.056	0.119
Liver	0.064	0.146
Lung	0.070	0.092
Muscle	0.053	0.135
Skin	0.037	0.141
Mean	0.059	0.121

Supplementary Table 5 | Estimated substitution rate data for gametologous gene pairs on the collared flycatcher Z and W chromosomes

Locus	Position on Z (bp)	Stratum	Pair d_S	NRW		Z chromosome	
				d_S	d_N/d_S	d_S	d_N/d_S
SMAD7	1397439	young	0.418	0.136	0.102	0.300	0.007
CTIF	1499112	old	0.493	0.182	0.218	0.403	0.070
SMAD2	1861091	-	0.231	-	-	-	-
C18orf25	2674578	young	0.366	0.110	0.265	0.228	0.216
ATP5A1	2739445	young	0.397	0.076	0.055	0.294	0.042
UBAP2	8306662	young	0.223	0.103	0.392	0.118	0.189
DCAF12	8430857	young	0.268	0.100	0.108	0.109	0.147
UBAP1	8471437	young	0.275	0.093	0.416	0.154	0.162
FAM219A	8589404	-	0.277	-	-	-	-
VCP	9497605	young	0.210	0.089	0.000	0.139	0.006
GOLPH3	10866720	-	0.324	-	-	-	-
ZFR	10975563	young	0.286	0.074	0.147	0.202	0.025
SUB1	11012552	young	0.170	0.050	0.071	0.074	0.122
NIPBL	12582107	young	0.189	0.060	0.064	0.106	0.062
PRKAA1	14086131	-	0.332	-	-	-	-
RPL37	14105142	-	0.176	-	-	-	-
ZNF131	14898488	young	0.205	0.074	0.294	0.136	0.138
SNX18	17817091	young	0.200	0.146	0.175	0.072	0.048
MIER3	18857934	-	0.198	-	-	-	-
ZSWIM6	20574573	-	0.153	-	-	-	-
KIF2A	20905811	old	0.187	0.076	0.188	0.140	0.098
SREK1	22411155	-	0.292	-	-	-	-
MRPS36	23541133	young	0.237	0.176	0.335	0.053	0.561
COL4ABP	25692644	-	0.226	-	-	-	-
TNPO1	26868143	-	0.193	-	-	-	-
MAP1B	27144807	young	0.283	0.105	0.670	0.178	0.148

RFX3	28550597	old	0.262	0.146	0.197	0.105	0.036
CDC37L1	29009066	old	0.551	0.248	0.343	0.276	0.123
CHD1	30131651[a]	old	0.400	0.132	0.164	0.241	0.032
RASA1	30131651[a]	old	0.330	0.137	0.073	0.189	0.020
GNAQ	36520702	old	0.255	0.109	0.028	0.207	0.029
novel2	36685973[a]	-	0.285	-	-	-	-
HNRNPK	36685973[a]	old	0.324	0.132	0.025	0.200	0.011
SPIN	40533877	old	0.229	0.098	0.039	0.113	0.000
NFIL3	41771706	old	0.340	0.119	0.699	0.216	0.052
HINT1[b]	42292467	old	-	-	-	-	-
KCMF1	44466461	old	0.314	0.221	0.155	0.169	0.113
RNF38	46376861	old	0.385	0.109	0.609	0.269	0.068
FEM1C	53787080	old	0.365	0.150	0.144	0.192	0.057
ZFAND5	57062641	-	0.191	-	-	-	-
ZNF462	58249660	old	0.553	0.236	0.275	0.351	0.050
ARRDC3	64237725	old	0.318	0.152	0.250	0.198	0.048
CKMT2	68100720	old	-	0.137	0.129	1.348	0.029
novel1	68591987	old	-	0.097	0.265	1.692	0.063

Lineage-specific rates were not estimated for gene trees with a bootstrap support <0.7. [a] The location of these genes is approximated since the corresponding scaffolds have not been ordered by confidence. [b] multi-copy gene and therefore excluded from substitution rate estimation.

Supplementary Table 6 | Number of segregating sites and levels of nucleotide diversity (π) of NRW and autosomes, respectively, in different flycatcher populations.

Species/population	W chromosome		Autosomes
	# SNPs	π	π
Collared flycatcher			0.00397
Italy	543	0.000061	
Hungary	597	0.000056	
Czech Republic	435	0.000038	
Sweden (Öland)	268	0.000047	
Pied flycatcher			0.00320
Spain	189	0.000044	
Czech Republic	596	0.000067	
Sweden	486	0.000069	
Sweden (Öland)	462	0.000062	
Semi-collared flycatcher	167	0.000042	0.00297
Atlas flycatcher	337	0.000062	0.00307

Supplementary Table 7 | Lineage-specific derived substitutions in coding sequences of NRW genes

Species	Synonymous	Non-synonymous
Collared flycatcher	10	9
Pied flycatcher	4	5
Atlas flycatcher	12	10
Semicollared flycatcher	11	6

Table specifies fixed substitutions unique to each lineage. Substitutions were polarized using red-breasted flycatcher as outgroup, based on parsimony.

Supplementary References

1 Li, H. & Durbin, R. Fast and accurate short read alignment with Burrows-Wheeler Transform. *Bioinformatics* **25**, 1754-1760 (2009).

2 Harris, R. S. Improved pairwise alignment of genomic DNA. *PhD thesis, Pennsylvania State University* (2007).
